# Encapsulated Peptides and Proteins with an Effect on Satiety

**DOI:** 10.3390/nano13071166

**Published:** 2023-03-24

**Authors:** Rafael O. de A. Costa, Thaís S. Passos, Eloyse Mikaelly de S. Silva, Nicolle Caroline S. dos Santos, Ana Heloneida de A. Morais

**Affiliations:** 1 Biochemistry and Molecular Biology Postgraduate Program, Biosciences Center, Federal University of Rio Grande do Norte, Natal 59078-970, RN, Brazil; 2 Nutrition Postgraduate Program, Center for Health Sciences, Federal University of Rio Grande do Norte, Natal 59078-970, RN, Brazil; 3 Nutrition Course, Center for Health Sciences, Federal University of Rio Grande do Norte, Natal 59078-970, RN, Brazil

**Keywords:** peptide, protein, obesity, encapsulation, anti-obesity agents

## Abstract

The world scenario has undergone a nutritional transition in which some countries have left the reality of malnutrition and now face an epidemic of excess body weight. Researchers have been looking for strategies to reverse this situation. Peptides and proteins stand out as promising molecules with anti-obesity action. However, oral administration and passage through the gastrointestinal tract face numerous physiological barriers that impair their bioactive function. Encapsulation aims to protect the active substance and modify the action, one possibility of potentiating anti-obesity activity. Research with encapsulated peptides and proteins has demonstrated improved stability, delivery, controlled release, and increased bioactivity. However, it is necessary to explore how proteins and peptides affect weight loss and satiety, can impact the nutritional status of obesity, and how encapsulation can enhance the bioactive effects of these molecules. This integrative review aimed to discuss how the encapsulation of protein molecules impacts the nutritional status of obesity. From the studies selected following pre-established criteria, it was possible to infer that the encapsulation of proteins and peptides can contribute to greater efficiency in reducing weight gain, changes in adipose tissue function, and lower hormone levels that modulate appetite and body weight in animals with obesity.

## 1. Introduction

Peptides and proteins are molecules with potential applicability in numerous sectors. The growth of this use accompanied technological advances, culminating in several applications, such as use in diagnostics, anti-aging, and cancer therapies, and the treatment of other diseases, such as obesity and diabetes mellitus [1,2,3,4,5].

From this perspective, the US Food and Drug Administration (FDA), from 2011 to 2016 [6], approved more than 60 new protein therapies. Given the importance of these peptides and proteins, there is already investment in the global market for drug synthesis based on peptides and proteins, which, according to Verified Market Research, was estimated at $26.98 billion in 2019 and is projected to reach $51.24 billion by 2027 [7].

Aiming at the treatment of obesity, according to Zizzari et al. [8], the most sold peptide drug to treat metabolic diseases is liraglutide (Victoza^®^), an analog of glucagon-like peptide (GLP-1), resistant to degradation by the gastrointestinal tract enzyme dipeptidyl peptidase 4 (DPP-4). This analog prolongs the half-life of GLP-1 and, consequently, its action [9,10].

However, despite the effectiveness of treatment with this drug, limitations prevent this molecule’s applicability in managing obesity. Since the efficacy of therapy can commonly be limited by adverse effects, especially those of a gastrointestinal nature, including nausea, vomiting, diarrhea, or constipation, another limitation is that treatment with GLP-1 analogs does not significantly increase energy expenditure [11,12].

Therefore, for treating obesity, it is interesting to search for alternatives since several medications have side effects causing damage that affects multiple tissues and organs. Among those listed, we highlight myocardial infarction, stroke, severe neuropsychiatric problems, insomnia, dizziness, and dry mouth [13,14,15,16]. Given the exposure to these undesirable effects, designing new interventions for delivering drugs and anti-obesity molecules is necessary to overcome the challenge of providing more effective and safer treatments.

In addition, oral therapy with peptides and proteins has many limitations, which can interfere with the active effect. During administration, the path of these molecules through the gastrointestinal tract faces numerous barriers, such as exposure to different pHs, degradation by proteolytic enzymatic action in the stomach or intestine, fermentation by bacteria, which can limit absorption, and physical barriers, such as the mucus layer of the goblet cells of the intestinal epithelium and others [17,18,19].

Thus, the targeted delivery of peptides and proteins is of great interest to the pharmaceutical industry, and encapsulating these actives of protein origin can ensure better stability of these molecules, protecting against the gastrointestinal tract environment, thus minimizing structural changes and losses and preserving biological functions and effectiveness [20].

Thus, encapsulation is an alternative to help improve the effectiveness of peptides or proteins used to treat conditions related to obesity. Studies show the potential of encapsulation to enhance several drugs’ actions when encapsulated [21,22,23,24].

Several materials are used to protect compounds or molecules intended for obesity therapy, and these carriers include polymer conjugates, hydrogels, microneedles, liposome systems, and many others [25].

The delivery system for peptides and proteins with different types of matrices promotes a series of changes in these molecules. The active’s electrostatic interactions with the biopolymers impact the system retention capacity and the resistance to physical and chemical conditions to which the proteins are susceptible, such as changes in conformation, which may cause a loss in biological activity. Another limitation of using these molecules is that the proteins have limited absorption in the gastrointestinal tract [26]. However, protein encapsulation systems overcome this limitation by increasing permeability in the cell membrane [20,27].

Given the above, it is necessary to demonstrate the importance of encapsulating peptides and/or proteins and point out the importance of this technology in developing new alternatives for treating obesity. Thus, aiming to answer the question “Do encapsulated peptides and proteins increase the effect on satiety, impacting weight gain in the state of obesity?”, this integrative review was developed [28].

This integrative review included preclinical trials studying the effect of encapsulated protein molecules for their role in satiety in a way that can impact weight gain in the state of obesity. We consider peptides and proteins, molecules with this character of natural or synthetic origin, as alternatives in treating obesity. 

There is a wide variety of studies in the literature using these molecules. Still, the clear majority do not provide information on how encapsulation can act on these peptides and proteins to achieve control of food intake, weight gain, and monitoring of hormonal parameters associated with obesity. In this review, we approach how encapsulation can be a strategy to improve the action of the studied molecules.

For this, an electronic search was carried out in the following databases: *Biblioteca Virtual de Saude* (VHL), SCOPUS, and Web of Science. Searches were based on keywords indexed in the Medical Subject Headings (MeSH): peptide, protein, obesity, encapsulation, and nanoparticles. The manuscripts were written in English, published in the last decade, and available in full in the cited databases. The title and abstract were analyzed for the study selection to be included in this review. Selection criteria included having peptides and proteins with activity/bioactivity in obesity and investigating their use in animals (rats or mice) with induced obesity. Articles were excluded when they did not cumulatively meet all inclusion criteria.

## 2. Encapsulation Methods and Techniques Used to Enhance the Effect of Peptides and Proteins on Obesity

**Figure 1:**
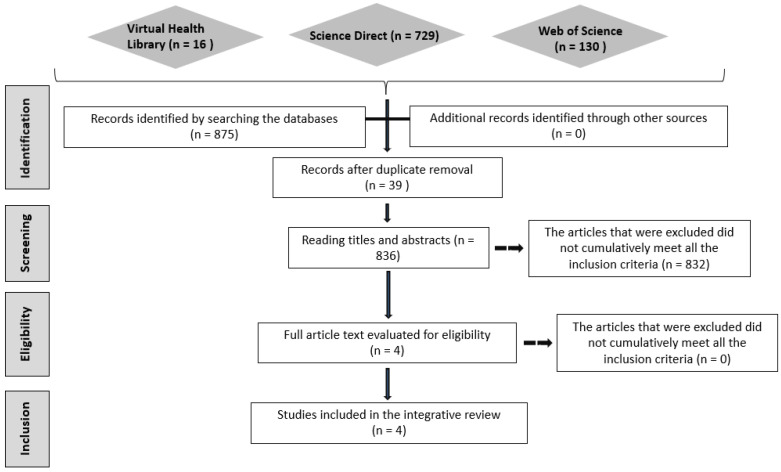
Article selection flowchart.

In the search carried out, according to the strategies mentioned above, of the 836 selected, comments, editorials, letters to the editor, theses, dissertations, conclusion studies, cross-repeated studies, and reviews were excluded (832) (Figure 1). Thus, only four articles related to the review focus met the selection criteria.

Among the studies, two were performed with peptides encapsulated in liposomes, with an in vivo study using mice with obesity induced by a high-fat diet [29,30]. For studies with proteins, two studies were found, in which the researchers used liposomes and polymeric nanoparticles, with an in vivo study in mice with obesity induced by a high-fat diet and a high-glycemic-index, high-load diet [31,32]. From these studies, it is possible to identify the limitations of using these molecules as candidates for their use in humans [18,19,26].

In this context, encapsulation appears as an alternative to enable the preservation of the encapsulated material, so peptides and proteins can increase their stability, reduce toxicity, as well as resist the physical and chemical conditions found in the environment [33,34].

**Figure 2:**
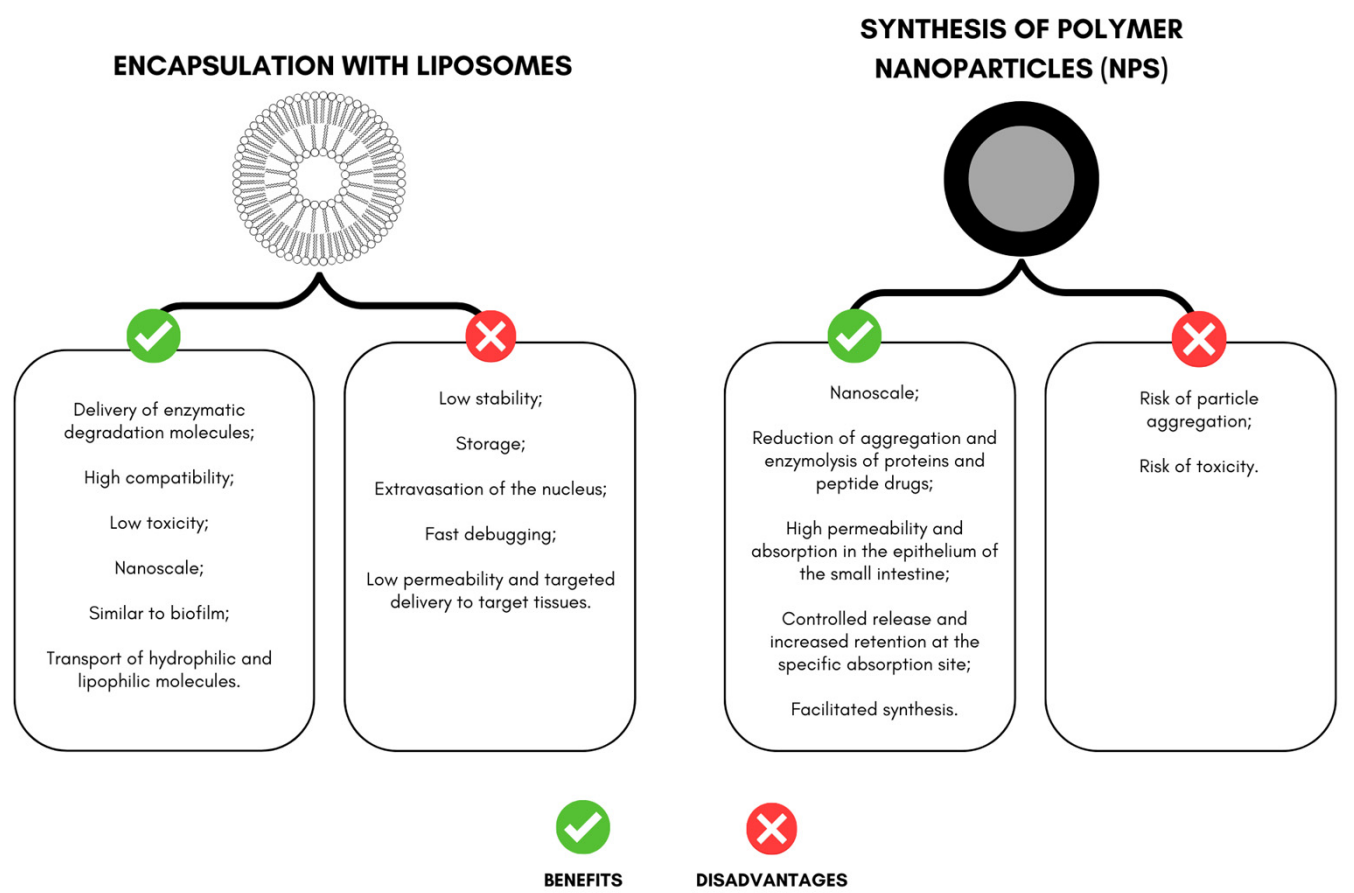
Advantages and disadvantages of encapsulation with liposomes and nanoparticles aimed at delivering peptides and proteins with effects on weight loss in experimental models.

The encapsulation of peptides and proteins comprises techniques that promote active protection, especially for those administered orally, and allow for release at the target site, targeting the action [22,23]. In this review, among the actives studied, synthesized peptides stand out, such as the PDBSN peptide (GLSVADLAE-SIMKNL) and the pro-apoptotic peptide (D (KLAKLAK)2, KLA), KLA) (Figure 2). For proteins, selected works include cytochrome C and trypsin inhibitors isolated from tamarind seeds.

Faced with the challenges of using protein molecules in particulate systems, the choice of polymers and techniques will directly impact the physicochemical characteristics, such as size, shape, functionality, stability, release profile, and increased bioactivity [35,36]. The main systems encapsulating peptides and proteins in the selected studies include liposomes and polymeric nanoparticles.

Liposomes are composed of a lipid bilayer, presenting a spherical shape with an aqueous core; they are also referred to as vesicles, and the size can vary from 25 nm to 2.5 μm [37]. In the literature, liposomes can be classified based on their size and number of bilayers as multilamellar vesicles (>500 nm) and unilamellar vesicles. Unilamellar vesicles can be further classified as large (LUV, >100 nm) and small unilamellar vesicles (SUV, <100 nm) [38,39].

This system has been used to deliver enzymatic degradation molecules with high compatibility and low toxicity. In addition, the biofilm-like structure can carry hydrophilic and lipophilic compounds [40,41]

Several studies have proposed liposomal formulations containing peptide and protein drugs to deliver different therapies, including infectious diseases, antitumor therapy, and treatment of allergic conditions, among other applications [42].

Despite liposome encapsulation’s advantages and diverse applications, it still faces problems related to stability, storage, active extravasation, rapid clearance, low permeability, and targeted transport to the target tissue [40,43].

Changes reduce these limitations in liposome structures, such as changes in lipid composition, surface coating, and addition of polymers or ligands, in addition to other technological strategies to face these limitations in applying liposomes [38,43].

The polyethylene glycol (PEG) covalent attachment to a molecule is known as “PEGylation.” This technique can extend to liposomes, peptides, carbohydrates, enzymes, antibody fragments, nucleotides, small organic molecules, and even nanoparticle formulations [44]. Modulating the liposomal composition can prevent clearance in the endoplasmic reticulum, promote mechanisms of resistance to enzymatic degradation, and improve mucous adhesion [38,45].

Polyethylene glycol polymers can influence the penetration capacity of the mucus to the PEG chains of Pluronic F127 on the surface of liposomes, facilitating the hydrophobic and electrostatic interactions of liposomes with mucins [46].

Shen et al. [30] performed liposome encapsulation to protect the PDBSN peptide, using PEG2000 to synthesize the formulation. Authors of other works highlighted the use of this polymer in liposomes, aiming to promote resistance to bile salts and improve insulin stability [47,48,49]. Despite the studies using insulin as an encapsulated peptide, for the present review, these studies were discarded because they did not address the application of this molecule in a delivery system in models of animals with obesity.

Even with reports of using liposomes to transport peptides and proteins for in vivo application, further studies are still needed to synthesize formulations capable of efficiently transporting molecules of protein origin for clinical application. As highlighted in this review, there are some challenges in using liposomes to promote the delivery of active components. Thus, it is necessary to understand the absorption mechanisms involved in active delivery through this encapsulation system. Using new technologies and methods can be an alternative to promote changes in the lipid composition, insert polymers to achieve new therapies, and enable liposomes to treat other diseases.

This review also evaluated the use of polymers in encapsulation systems to promote the protection of the active agent. In particulate systems, these components can be called wall materials, mainly referred to in the literature as shells, capsules, membranes, or matrices [50].

With the use of these polymers, it is possible to promote the protection of peptides and proteins from adverse conditions in the in vivo environment, such as exposure to pH, enzymatic action, and fermentation by bacteria, in addition to the physical barriers of the GIT, such as mucus [17,19].

Nanoparticles (NPs) can be synthesized using natural polymers, such as chitosan, alginate, gelatin, albumin, whey protein, and casein [51], or synthetic agents, such as silica, ceramics, and metallic oxides. This is interesting because it allows polymeric nanoparticles to be produced using emulsification, nanoprecipitation, ionic gelation, and microfluidics [52,53].

Thus, each material and technique can confer specific physical–chemical characteristics, influencing the particles’ size, shape, and functionality [54]. Examples of NPs include solid lipids, silver, gold, magnets, mesoporous silica, nanocrystals, carbon nanotubes, albumin and fullerene nanoparticles, and polymeric nanoparticles [52].

Some of the advantages mentioned in the literature for the delivery of drugs promoted by NPs containing proteins and peptides include reductions in aggregation and enzymolysis of proteins and peptide drugs in the gastrointestinal environment, increased trans-membrane absorption in the epithelium of the small intestine, changes in the distribution of the active in the body, facilitated synthesis, controlled release, and increased retention at the specific absorption site [55].

Costa et al. [32] investigated the encapsulation of TTI through nanoprecipitation in an organic solvent to obtain efficient polymeric particles. It was observed that the interaction of the active with the encapsulating agents increased the stability of the inhibitor at a neutral pH and potentiated the activity of the protein concerning the effect on weight reduction in Wistar rats with obesity.

The nanoprecipitation method is reportedly simple, easy, fast, cheap, and reproducible [56,57]. This technique comprises an organic phase introduced into the aqueous phase during nanoprecipitation. For the encapsulating agent and the active interaction to occur, the polymer and the water-miscible organic solvent, which must be miscible in the aqueous medium, make up the organic phase, leading to a diffusion effect [52,58].

To delay aggregation, the polymer must be insoluble in the aqueous solution, which may contain a surfactant, such as Tween [52,59,60]. Adding the organic phase to the aqueous phase with moderate agitation produces the particles. These particles are recovered by ultracentrifugation and then washed in water to remove surfactant residues. The organic solvent must be quickly evaporated, as occurs with ethanol, acetone, hexane, or methylene chloride, hardening the particles, which are later recovered via filtration, centrifugation, or lyophilization [52]. 

However, the disadvantages of polymeric NPs include an increased risk of particle aggregation and toxicity [61]. Only a few polymeric nanodrugs are approved by FDA and used in the clinic. Still, polymeric nanocarriers are being tested in several clinical trials (from 2011 to 2016) [6].

Thus, research has revealed delivery system techniques, especially for peptides and proteins, explaining how these materials can increase the stability and potentiate the bioactive action of these molecules. However, further studies are needed to enable the delivery of peptides and proteins by polymeric systems.

## 3. Encapsulated Peptides and Proteins with Effects on Obesity in Experimental Models

Numerous studies highlight the importance of encapsulating peptides and proteins to overcome their application difficulties [62,63]. Furthermore, this topic intends to highlight peptides and proteins in delivery systems, emphasizing details about the characteristics of wall materials, as well as the additional principal effects on obesity generated by the action of encapsulation (Table 1).

As previously stated, the interaction of the active with the delivery materials in the encapsulation of peptides and proteins can cause changes that will or will not potentiate the biological activity of the active component. Moreover, in vitro and in vivo models are necessary for understanding the applicability of peptides and proteins [64,65,66]. Thus, researchers have highlighted using encapsulated, nano-, or microencapsulated peptides and proteins on weight reduction, especially in an experimental model using animals with induced obesity. 

Shen et al. [30] investigated a peptide (PDBSN) with the ability to induce inhibition in the differentiation of adipocytes, in which the molecule acts in the activation of the AMP protein kinase activity pathway (AMPK). The researchers observed, in in vitro tests with human pre-adipocytes and stem cells derived from mouse adipose tissue (C57BL/6J), the influence of the peptide as a possible anti-adipogenic agent independent of cell proliferation or apoptosis.

Additionally, Shen et al. [30] encapsulated a peptide (PDBSN—(GLSVADLAESIMKNL)) in liposomes associated with two ligands (peptide directed to visceral adipose tissue and peptide of cellular penetration) and aimed to improve the stability and specificity of the active. This system was evaluated in mice with obesity induced by a high-fat diet (HFD). Treatment with the encapsulated peptide, despite not having a significant impact on food intake, caused a reduction in the weight of the animals (at a dose of 5 and 10 mg/kg) compared to animals treated with the control peptide (MNAVSLELADLGSKI)—at a dose of 10 mg/kg—with animals fed the HFD diet [30].

Finally, Shen et al. [30] explained that weight reduction was achieved by increasing the accessibility of the peptide in adipose tissue. This new function reduced fat mass, with a consequent decrease in weight gain in mice fed HFDs. The researchers also described other effects observed in the animals, such as improved glucose, adiponectin, and reductions in high levels of leptin. Improvements in inflammatory factors, such as IL-6 and TNF-α, were also demonstrated, and both were reduced by treatment with the encapsulated peptide [30].

A study by Hossen et al. [29] used nanoparticles containing prohibitin. This peptide is a mitochondrial chaperone, belonging to a family of proteins with hydrophobic residues. The peptide nanoparticles allowed for chemical interaction and organization in the spaces between the membranes [29].

The nanoparticles contained the pro-apoptotic peptide, (D(KLAKLAK)2, KLA), called adipotide, which binds specifically to prohibitin on the endothelial cell surface of fat white vessels (FWVs). As a delivery system, PEGy-sided liposomes modified by PTP (prohibitin targeting peptide) were synthesized [29]. PEG is a long polyethylene glycol spacer that reduces steric hindrance associated with ligand–receptor interactions. A short PEG polymer was also used in this study as a surface biostabilizer to accelerate the circulation time of the peptide in the plasma. This formulation was prepared using the reverse phase evaporation (RPE) liposome production method [29].

According to Hossen et al. [29], encapsulation enhanced peptide delivery. A dose three-times lower than the encapsulated molecule reduced the body weight of animals with obesity. In parallel, there was also a reduction in serum levels of leptin and adiponectin, hormones generally deregulated in obesity. Furthermore, there was an anti-obesity effect related to the complications of obesity, such as improvement in macrophage infiltration into adipose tissue and ectopic fat deposits in the liver and muscle [29].

Cytochrome C (CytC) is a small, highly degradable heme protein associated with the inner mitochondrial membrane, and it has apoptotic action by activating caspase [39]. In a study by Hossen et al. [31], CytC encapsulation was proposed to exert its function in white adipose tissue as a therapy to treat obesity. By encapsulating CytC, it showed greater stability and potentiated its effects on obesity in animals [31]. A delivery system was also used in PEGylated liposomes, modified by prohibitin targeting peptide (PTP). According to Hossen et al. [31], encapsulation improved the state of obesity in animals in a dose-dependent manner, preventing weight gain, increasing serum leptin, and leading to apoptosis in adipocyte endothelial cells.

Given the limitation in the use of proteins, considering the possibility of hydrolysis in the acidic and proteolytic environment of the stomach, in addition to the low permeability through the intestinal epithelium, when administering proteins with bioactive properties orally, using protection techniques such as encapsulation becomes a viable strategy to maintain or even enhance the activity of these molecules [67].

Trypsin inhibitors are proteins and may be an alternative to prolong the action of hormones, acting as secretagogues of cholecystokinin (CCK), a sacietogenic hormone that acts in the short term on food intake [68,69,70,71]. However, increasing the time of action on CCK could have a more powerful impact on food consumption and, consequently, weight loss.

Costa et al. [32] nanoencapsulated the tamarind trypsin inhibitor (TTI) using a combination of chitosan and whey protein (ECW) wall materials and obtained stable nanoparticles, showing solid chemical interactions between the active and the encapsulating agents. ECW was administered to Wistar rats with obesity induced by a high-glycemic-index and high-glycemic-load (HGLI) diet. TTI and ECW were evaluated for several zoometric parameters, including weight variation. The data showed greater weight loss, and this reduction was significant in the ECW-treated group compared to the TTI-treated group.

The authors highlighted that the reduction in the weight of animals points to a new action of the inhibitor on obesity [32]. This effect was only achieved in eutrophic animals treated with unencapsulated TTI [69]. However, in some studies on obese rats treated with non-encapsulated TTI, weight loss was not observed [72,73].

Among the studies presented, the variety of effects promoted by peptide and protein encapsulation on the state of obesity and the most diverse mechanisms and modifications that occur in these molecules with the encapsulation can be perceived.

## 4. Main Effects Potentiated by the Encapsulation of Peptides and Proteins

**Figure 3:**
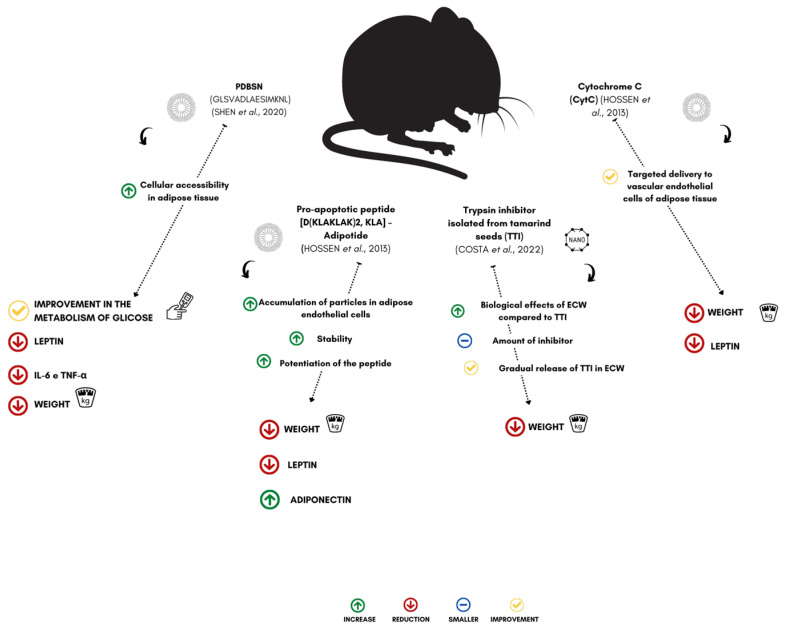
Effect of encapsulated peptides and proteins on weight loss in experimental models. PDBSN (peptide GLSVADLAESIMKNL), KLA-Adipotide (pro-apoptotic peptide (D(KLAKLAK)2), TTI (trypsin inhibitor isolated from tamarind seeds), CytC (cytochrome C), IL-6 (interleukin-6), and TNF-α (tumor necrosis factor alpha) [29,30,31,32].

Encapsulation through micro- and nanosystems has become an innovative and promising approach for changing the native properties of molecules and active compounds against the environment during processing, storage, handling, delivery, and improved acceptance as a product [74]. This provides greater solubility, stability, bioavailability, and changes in sensory characteristics (odor and taste). For peptides and proteins, promoting a controlled release system can reduce potential exposure to side effects, achieve adequate concentrations of the active in the bloodstream, and increase the therapeutic effect, among other advantages [35,75]. In this review, given the selected studies, it was found that encapsulation enhanced the therapeutic effects of peptides and proteins (Figure 3).

Hossen et al. [29] observed that the encapsulation of a pro-apoptotic peptide (D (KLAKLAK)2, KLA), adipotide, in nanoparticles targeting prohibitin (an adipose vascular marker) (PTNP) could reduce the body weight of animals with obesity, treated (mice of the C57BL/6J lineage) under the effect of a significant decrease in serum leptin levels, in parallel with an anti-obesity effect on adipose tissue.

Kolonin et al. [76] used a peptide (KGGRAKD) with the capacity to bind specifically to prohibitin on the endothelial cell surface in white adipose tissue vessels, aiming to develop therapies for the treatment of obesity. This molecule was associated with another peptide, the cell-death-inducing peptide (KLA), for delivery to adipose tissue, where it could promote weight loss in mice with obesity (C57BL/6J strain mice).

Despite this finding, Hossen et al. [29] carried out, in their study, the encapsulation of the pro-apoptotic peptide ((D (KLAKLAK)2, KLA), adipotide, to understand the effects of therapeutics directed by nanoparticles on obesity in treated animals. In this study, the authors highlighted the advantages of the KLA-PTNP system compared to the bioconjugate system, adipotide, without the incorporation of nanoparticles, in treating diet-induced obesity in mice of the C57BL/6J lineage.

The results showed that the KLA-PTNP system potentiated the action of the encapsulated peptide, with a dose three-times lower, exerting a greater therapeutic effect on weight loss. The data also showed that mice treated with particles substantially reduced body weight due to decreases in adipose tissue and the influence of adipocyte hypertrophy.

In the case of the non-encapsulated molecule, there was an insignificant decrease in the size of the adipocytes. Nanoparticles can reduce the ectopic fat content in the liver and muscle tissue through fat oxidation induced by increased adiponectin [29]. However, adipotide treatment also significantly reduced ectopic fat accumulation in the liver and muscle, but the therapeutic efficacy of KLA-PTNP was considerably higher. The nanoparticles acted as a micro reservoir, consisting of fractions released from the peptide, allowing them to be delivered in a controlled manner over a long period [29].

Shen et al. [30] identified a new peptide derived from the A chain of L-lactate dehydrogenase, PDBSN, from intracellular adipose tissue peptides, preventing adipogenic differentiation of pre-adipocytes without impacting cell proliferation or apoptosis. The authors highlighted that this peptide could repress adipogenic differentiation by activating the AMPK signaling pathway. Therefore, they proposed peptide encapsulation (PDBSN) to develop an effective strategy to control adipogenesis, aiming at controlling obesity and metabolic disorders associated with excess adiposity.

Encapsulation of the peptide in liposomes, combined with two ligands, improved stability and specificity. This new molecule promoted improvements in obesity induced by a high-fat diet and improved metabolism homeostasis (glucose metabolism, hepatic steatosis, and dyslipidemia) [30]. During the treatment, the animals that received the liposomes containing the PDBSN were monitored for their accessibility by fluorescence in the organs in the first 24 h. A higher concentration of the peptide was observed in the liver, brown adipose tissue, subcutaneous fat, visceral fat, epididymal fat, and skeletal muscle. Compared to the control group, PDBSN labeled with fluorescein isothiocyanate (FITC), very little fluorescence was identified in the liver, suggesting it was rapidly eliminated in vivo [30]. For this trial, the authors suggested that these liposome modifications improved the stability and specificity of PDBSN in adipose tissue, especially visceral fat (almost five-times greater than in other adipose tissues).

The HFD-induced obesity mouse model was adopted to determine the role of PDBSN in vivo on weight gain. Male mice, strain C57BL/6, six to eight weeks old, were fed a high-fat diet for eight weeks. The intervention with intravenous injections of liposomes with the PDBSN (5 mg/kg and 10 mg/kg) was administered once a week. Meanwhile, the control peptide (scramble) encapsulated in liposomes was simultaneously injected as a control. Thus, compared to the control group fed a high-fat diet, weight gain in the PDBSN-treated group (10 mg/kg) was significantly reduced, regardless of dietary intake [30].

After animal sacrifice, ref. [30] observed a significant reduction in subcutaneous, visceral, and epididymal adipose mass in mice treated with 10 mg/kg of PDBSN. In comparison, 5 mg/kg of peptide affected the fat mass moderately. Interestingly, the volume and weight of visceral fat significantly decreased after treatment with the peptides, which may be due to the greater accessibility of PDBSN in visceral adipose tissue, indicating a dose-dependent function of the peptide in adipose tissue.

Treatments with antiangiogenic drugs have shown promise for weight reduction and adipose tissue loss in various models of obesity. Cytochrome C (CytC) is a multifunctional protein, acting as an electron carrier in the mitochondrial electron transport chain, being indispensable for organisms that need the production of ATP. In addition, under conditions of cellular stress, CytC is released from the mitochondria to the cytosol, which interacts with apoptotic protease activating factor 1 (Apaf-1) to form the apoptosome, leading to activation of caspase-9 and the caspase cascade, with consequent cell death [77,78].

Due to the short half-life, in vivo, due to external inactivating effects (such as enzymatic degradation), prolonging the time for the target moieties to efficiently interact in the desired area of action and promote delivery to the site of action (endothelial cells of the white tissue adipose) becomes indispensable. Thus, pharmacological manipulation through the targeted nanoparticulate system offers an attractive therapeutic route for effectively managing obesity [31].

Faced with this apoptotic activity of CytC and the limitations of using this protein, [31] intended to deliver this molecule with the prohibitin-targeted nanoparticle system (PTNP) to develop an effective and safe anti-obesity therapy. Thus, the encapsulation promoted an increase in the activity of the protein in vitro. Treatment with various doses of PTNP with CytC (3, 6, and 12 µg/ml of CytC) induced activation of caspase 9, indicating apoptosis in primary endothelial cells isolated from murine adipose tissue (pcEC-IWAT) in a dose-dependent manner. On the other hand, this action was not evidenced in the control groups (free CytC at the same doses—3, 6, and 12 µg/ml, and cells treated with placebo).

The in vivo study evaluated whether the targeted delivery of CytC had any beneficial effect in preventing obesity. Animals received a high-fat diet (HFD) at three-day intervals for 30 days with intravenous injections of 6, 1.2, and 0.25 mg/kg of the CytC-PTNP system. The percentage increase in body weight for HFD-fed mice significantly decreased when treated with the CytC-PTNP system compared to the HFD control group, with a dose-dependent effect observed [31].

Furthermore, the obese animals treated with the CytC-loaded PTNP system did not decrease cumulative energy intake compared to untreated animals (HFD and normal diet). Despite this result, the role of protein encapsulation under the effect of adipose tissue (epididymal subcutaneous tissue) was investigated during the 30 days of treatment. The results showed that the CytC-PTNP system could reduce the increase in fat mass [31].

Serum biochemical parameters were investigated to evaluate the anti-obesity effect of treatments administered to animals with obesity. The HFD-fed control group had high levels of leptin, while the levels for the normal-diet-fed control group were maintained at normal levels. Treatment with CytC nanoparticles reduced serum leptin levels in a dose-dependent manner [31].

Studies with trypsin inhibitors present in tamarind may be an alternative for treating obesity. Studies with this molecule showed promising results in inflammation, metabolic parameters, and reductions in food intake, with a consequent reduction in weight gain only in eutrophic animals [69]. It is known that the satietogenic action of trypsin inhibitors is related to the increase in plasma CCK levels, leading to a rise in short-term satiety [68,69,70,71].

Although the isolated and purified trypsin inhibitor did not significantly affect the weight of obese animals [72,73], evaluating the action of this nanoencapsulated molecule is of great importance since the TTI can enhance its effects and achieve weight reduction in obese mice. This suggests that a particulate system can promote targeted delivery, greater protection, and stability, allowing for sustained and efficient release, and may be a resource to enhance and control the action of this protein in the release of CCK to promote changes in the nutritional status of obesity in the studied animals.

In the literature, it has been reported that encapsulated TTI (ECW) administered in an experimental model of diet-induced obesity promoted an improvement in carbohydrate metabolism, reduced glycemia, and improved liver parameters [79,80].

Given the potential evidenced by ECW in an in vivo model, understanding the role of this new molecule compared to non-encapsulated TTI on the satietogenic effects in obesity becomes necessary. This review describes a recent study by Costa et al. [32], who encapsulated TTI with chitosan and whey protein to evaluate its action on the consumption and weight of animals fed a high-glycemic-index and -glycemic-load (HGLI) diet.

In this study, comparing TTI and ECW, the authors pointed out a new action of the inhibitor since, with a dose ten-times lower than the bioactive dose of TTI, the animals had a greater effect on weight loss when treated with ECW. At the same time, TTI did not show such an effect. Although inconclusive, the authors suggested that the TTI was partially released from the particle throughout the treatment, which could sustainably act on CCK activity and, consequently, affect food intake and weight loss.

Finally, peptides and proteins intended for the treatment of obesity, when encapsulated, more efficiently affect reductions in weight gain, changes in the function of adipose tissue, such as the accumulation of adipose mass in the peripheral region, in addition to decreases in hormone levels that modulate appetite and body weight (Figure 3). However, there is still a limited number of studies that show the mechanisms of how encapsulation can enhance the bioactive activities of protein components and how these factors can be efficient in controlling obesity and its associated complications. Some studies have shown that encapsulation can be a viable technological alternative for applying these molecules in the gastrointestinal tract.

## 5. Mechanisms Related to the Effects of Peptides and Proteins on Obesity Promoted through Encapsulation

Industry is increasingly interested in seeking new strategies to promote the controlled release of bioactive molecules, aiming to identify and characterize new compounds and molecules that can be used as drugs, functional food ingredients, or nutraceuticals [81].

Maintaining the stability of molecules and bioactive compounds has been challenging, as they are susceptible to changes in functionality due to exposure to oxygen, light, heat, pH variation, and water. Some of these factors limit the shelf life and bioavailability of the application of these molecules [82]. In addition, since they are preferably intended and administered orally, these molecules and bioactive components are subjected to intestinal metabolism, causing a transformation in their chemical structure and changes in their biological functions. Therefore, as previously mentioned, it is advantageous to guarantee stability in the gastrointestinal tract and allow for a controlled release in the target tissue [83].

It is known that proteins and peptides can be encapsulated using several techniques. Based on this, these molecules can be delivered from micro- or nanoparticles. Therefore, the interest in encapsulated bioactive molecules depends on the possibilities of modifying physicochemical properties, for example, overcoming solubility incompatibilities, increasing stability, delivery at the site of interest, and controlled release, among others [84,85].

Given the articles selected for the present study, despite the encapsulation of peptides and proteins modifying the stability and enhancing the action of these molecules, the authors should have highlighted the mechanisms attributed to the encapsulation of the actives in question.

Implementing new technologies for delivering protein molecules is an area of increasing exploration for treating numerous conditions, including obesity. These molecules may become future candidates due to their specificity, low toxicity, and regulatory action in the pathophysiology of diseases such as diabetes [86,87].

Recent studies highlight that protecting peptides and proteins leads to several physicochemical alterations, which can help reduce the impact of oral administration, such as enzymatic degradation, instability in the gastric environment, low penetration of the intestinal membrane, short plasma half-life, and the tendency to undergo aggregation, adsorption, and denaturation [20,87,88].

Although the studies presented in this integrative review do not explore the interaction mechanisms of the actives and polymers, the physicochemical characteristics of peptides and proteins are essential in determining their encapsulation properties. Thus, these molecules can be attracted by interactions with anionic biopolymers (such as alginate and carrageenan) or cationic biopolymers (such as chitosan or polylysine). Alterations in electrostatic interactions between polymers are often used to modify retention, release, size, and bioactivity properties [20,50,89].

Despite the limitations of the oral use of peptides and proteins, there are several methods to increase the stability and delivery of these molecules. Some examples highlight using an enteric coating, enzyme inhibitors, permeation enhancers, proteinylation, glycosylation, PEGylation, and synthesis of nanoparticles and microparticles [87,88,90].

Of the selected articles, two papers highlighted using PEGylation and permeation enhancers in the form of peptides. These changes in the system increased the stability of the encapsulated molecules and the targeted delivery to the target tissue, with a consequent increase in the therapeutic effect.

The scarcity of works showing the interaction mechanisms of the active and the encapsulation in the increase in bioactivity, stability, and additional effects, especially in studies with applications in obesity, is a limiting factor in the literature. The limitations found in the studies selected for this review are a need for more information about the safety and clearance/accumulation of the micro- and/or nanocomplexes that could help elucidate, with greater clarity, the safety and effectiveness of using these molecules to validate their therapeutic use. Given these findings, further studies are needed to deepen knowledge of peptides and proteins in clinical practice, especially in treating obesity and its associated conditions. 

## 6. Perspectives and Limitations of the Use of Encapsulated Peptides and Proteins as Therapeutic Agents

With the emergence of new technologies, peptides and proteins have been alternatives for treating various diseases. These innovative methodologies allow these molecules to overcome limitations, especially in the in vivo environment, such as low half-life, rapid degradation by peptidases, and low absorption by mucous membranes (such as the small intestine) [55,91].

Given these administration limitations and low oral bioavailability, peptides and proteins are mainly administered parenterally, which is inconvenient and sometimes even painful and risky. Continuous long-term applications can pose a major medication adherence challenge, including pain, aversion to injections, concerns about needle size, and local irritation [92]. Several routes have been developed to promote the controlled release of peptides and proteins, such as oral, nasal, ophthalmic, buccal, and transdermal. Even in the face of variability, oral administration is the most attractive region due to greater safety and adherence to treatment [93].

In addition, oral therapy with peptides and proteins has many limitations, which can interfere with the effect of the active. During administration, the course of these molecules through the gastrointestinal tract faces numerous physiological barriers, such as exposure to different pHs, degradation by proteolytic enzymatic action in the stomach or intestine, fermentation by bacteria, which can limit absorption, in addition to physical barriers, as the mucus layer of the goblet cells of the intestinal epithelium and others [18,19].

The effects of extreme pH variations in these molecules can lead to increased interactions with electrostatic repulsions, promoting conformational changes crucial for biological function. The pH of the GI tract, gastric fluids (pH 1–3), duodenum (pH 6–6.5), and colonic pH (5.5–7.0) can have different effects on peptide and protein interactions. PH variations impact the electrostatic interactions of these molecules, and in lower ranges, they increase the positive charge. At higher pH, there are alterations that cause electrostatic repulsion, modifying the structure and limiting the functional part of the bioactive molecule [18,19].

In addition to this effect of pH variation, enzymes can remarkably impact the binding of protein molecules. Encapsulating these actives of protein origin can guarantee better stability for these molecules, protecting them against the proteolytic environment of the gastrointestinal tract, as proteins with bioactive functions can lose their activity due to changes in their three-dimensional structure or due to hydrolysis in undesirable situations. This would significantly affect biological function and effectiveness [20].

In the stomach, pepsin is responsible for the cleavage of peptides and proteins at the level of the intestine with the action of peptidases on the brush border villi and proteases in the portion of the duodenum, small intestine, and colonic region. Among the enzymes are trypsin, carboxypeptidase A/B, elastase, and chymotrypsin, which degrade almost all amino acids. Most proteins are affected by luminal enzymes, amino peptidase (A/-N/-P/-W), γ-glutamyl-transpeptidase/-carboxypeptidase, endopeptidase, and enteropeptidase. The enzymatic breakdown of these molecules generates by-products that remain smaller peptides unable to lead to therapeutic effects [86,88,91].

In addition to chemical barriers, physical ones affect the absorption of peptides and proteins. The main difficulty coping with these molecules is the mucus barrier, composed of elements that prevent oral protein components’ transport. The mucus barrier is composed of mucin, formed by a network of disulfide bridges forming a resistant viscoelastic gel. They interact with proteins through electrostatic interactions, van der Waals forces, hydrophobic interactions, and hydrogen bonds, making them difficult to absorb [86,88,91]. Another physical challenge for proteins and peptides to reach the surface of enterocytes is the glycocalyx, an acidic fibrous coating consisting of sulfated mucopolysaccharides, a difficult task for those molecules, with a molecular weight greater than 5 kDa [86].

Thus, encapsulation is an alternative to help improve the effectiveness of peptides or proteins used to treat conditions related to obesity. Studies show the potential of encapsulation to enhance several drugs’ actions for encapsulation [21,22,23]. Several materials are used to protect compounds or molecules intended for obesity therapy, and these carriers include polymer conjugates, hydrogels, microneedles, liposome systems, and many others [25].

The delivery system for peptides and proteins with different matrices (or encapsulation) promotes a series of changes in these molecules. The electrostatic interactions of the active with the biopolymers impact the retention capacity and the resistance to physical conditions and chemicals, to which proteins are susceptible, such as changes in conformation, with a loss of biological activity.

Given the above, one can see the importance of encapsulating peptides and/or proteins and pointing out the importance of this technology in developing new alternatives for treating obesity.

## 7. Conclusions

Even in the face of the challenges related to the use of peptides and proteins as promising candidates for the treatment of obesity, the encapsulation of these molecules can be a valid strategy for the development of new therapies to reduce the limitations of the use of proteinaceous substances in vivo and, thus, achieve the ideal treatment to delay weight gain.

With this review, it was possible to reveal a limited number of studies addressing the treatment of peptides and proteins, compared to the same encapsulated, for controlling food intake, weight gain, and monitoring hormonal parameters associated with obesity. The different therapeutic effects of encapsulated actives and the anti-obesity impact already evidenced in the scientific literature make encapsulation an advantageous method to overcome the limitations of the exogenous and endogenous environment. Therefore, further studies are needed to deepen knowledge in the area and to elucidate all the properties of encapsulation of peptides and proteins to obtain sufficient information validating their use in the treatment of obesity and its complications.

## Figures and Tables

**Table 1:** Characterization of studies carried out with encapsulated peptides or proteins with effects on weight loss in experimental models.

Reference	Peptide or Protein	Encapsulating Agent	Encapsulation Efficiency (EE) and Particle Diameter	In Vivo Study (Animal Model)	Obesity Model and Number of Animals	Intervention and Peptide Dosage (mg/µg)	Effects of Encapsulation	Effects on Obesity	Prospects for Action in the Control of Obesity
Hossen et al. [29]	Pro-apoptotic peptide [D(KLAKLAK)2, KLA]—Adipotide	PEGylated liposome modified by PTP (prohibitin-targeting peptide), referred to as prohibitin-targeted nanoparticle (PTNP)	9.4% e 109.2 (±7.8) nm Recovery rate (%) = P1/P0 × 100, where P0 = FI of total KLA inserted in the system, P1 = FI of encapsulated KLA.	Male C57BL/6J mice, six weeks old.	High-fat diet (HFD)-induced obesity. A total of 12 animals were used.	Encapsulated peptide (1 mg/kg KLA-PTNP), unencapsulated peptide (3 mg/kg adipotide), particles without peptide (control) (0.2 mmol/kg E-PTNP) at 3-day intervals for 30 days. In comparison, another group remained untreated and served as an untreated control.	Greater accumulation of particles in the adipose endothelial cells of animals and greater stability and potentiation of the peptide. Weight gain in the group treated with the encapsulated peptide (3 times lower dose) was about 3 times lower compared to the group treated with the non-encapsulated peptide.	At the end of the experiment, the body weights of mice treated with encapsulated peptide were significantly lower than baseline weights (14% reduction), with a decrease in adipose tissue and adipocyte hypertrophy. There was a reduction in serum leptin levels and increased serum adiponectin.	The encapsulated peptide can potentially reduce ectopic fat content in the liver and muscle tissue through adiponectin-induced fat oxidation. In addition to the potential to reduce weight gain in obese mice by destroying adipose vessels.
Shen et al. [30]	Peptide—PDBSN (GLSVADLAESIMKNL).	Liposome	63.14% e 165.9 nm The calculation was not informed	Male C57BL/6J mice, six weeks old.	High-fat diet (HFD)-induced obesity for eight weeks. A total of 32 animals were used.	Encapsulated peptide (5 mg/kg and 10 mg/kg) was administered weekly for eight weeks. As a control, the control peptide (scrambled–Scr) (10 mg/kg) encapsulated in liposomes was simultaneously injected once a week.	High accessibility (or cell penetration), stability, and potentiation of peptide bioactivity.	The peptide encapsulated in liposomes affected weight loss with a significant decrease in subcutaneous, visceral, and epididymal adipose mass, with improvement in glucose metabolism (glucose tolerance and insulin sensitivity). In addition, they showed an increase in adiponectin gene expression, and a reduction in leptin gene expression, with a significant decrease in the expression of inflammatory markers (IL-6 and TNF-α).	PDBSN could decrease fat mass and delay weight gain in HFD-fed mice.
Hossen et al. [31]	Cytochrome C (CytC)	PEGylated liposome modified by PTP (prohibitin-targeting peptide), referred to as prohibitin-targeted nanoparticle (PTNP)	15.23% e 120.1 (± 15. 4) nm Recovery rate (RR): %RR = P1/P0 × 100, where P0 = optical density of the total CytC used in the nanoparticle (free + encapsulated), P1 = optical density of CytC (encapsulated).	C57BL/6J mice, male, six weeks old.	HFD-induced obesity. A total of 15 animals were used.	Treatment with encapsulated protein (6, 1.2, and 0.25 mg/kg) at 3-day intervals for 30 days. As a control, treatment with particles without the peptide (0.1 mmol/kg).	The authors suggested that encapsulation promoted targeted and controlled delivery to vascular endothelial cells of adipose tissue in mice.	Intravenous treatment from CytC-containing particles resulted in a substantial reduction in obesity in mice, as evidenced by the prevention of an increase in body weight, being dose-dependent. In addition to the decrease in serum leptin levels, also in a dose-dependent manner.	TNF-α attenuation, but without significant difference.
Costa et al [32]	Trypsin inhibitor isolated from tamarind seeds (TTI)	Purified chitosan and whey protein isolate	95.19% e 109 nm (±6.7) nm Encapsulation efficiency (EI): EI (%) = (TTI in particles/total TTI used) × 100	Wistar rats, male	High glycemic index and glycemic load (HGLI) diet for 17 weeks. A total of 25 animals were used.	The encapsulated protein was administered 1 mL of ECW at 12.5 mg/kg gavage/day. As a control, treatment with non-encapsulated TTI (25 mg/kg) for ten days.	The authors suggested that in TTI, due to nanoencapsulation, a smaller amount of the inhibitor promoted interesting biological effects, being more potent in weight loss.	Treatment with the encapsulated inhibitor (ECW) showed a greater impact on the weight of the animals due to the potentiation of the inhibitor’s action.	The results suggest that ECW, releasing TTI sustainably, prolongs the action of CCK, directly impacting food consumption with consequent weight loss.

## Data Availability

The data presented in this study are available on request from the corresponding author.
